# The role of cervids (C*ervus elaphus*) in the ecobiology of some tick- borne diseases

**DOI:** 10.1186/1756-3305-7-S1-P2

**Published:** 2014-04-01

**Authors:** V Mircean, Z Kalmar, M Mircean, A Györke, E Vitos, MO Dumitrache

**Affiliations:** 1 Department of Parasitology and Parasitic Diseases, University of Agricultural Sciences and Veterinary Medicine Cluj-Napoca, Calea Mănăştur 3-5 Cluj-Napoca 400372, Romania; 2 Department of Internal Medicine, University of Agricultural Sciences and Veterinary Medicine, Calea Mănăştur 3-5, Cluj-Napoca, 400372, Cluj, Romania

## 

The maintenance of tick-borne pathogens in nature follows a variety of patterns that are always involving ticks, reservoir hosts and pathogens. The red deer (*Cervus elaphus*) is the fourth-largest deer species and inhabits most of Europe in a wide variety of habitats. In the last 50 years the red deer (*Cervus elaphus*) population remained constant in Romania, with around 30,000 individuals and no significant fluctuations registered. These animals are one of the preferred hosts for *Ixodes ricinus*, a tick with vectorial capacity for a wide range of pathogens; they are reservoir hosts for many tick-borne diseases; due to their movement from sylvatic to synantropic environment and back they can influence the spreading of both ticks and pathogens. In Romania, studies about the epidemiology of tick-borne diseases in wild animals, eminently in red deer are limited. In this frame, our study aimed to describe the diversity of ticks that are parasitizing *Cervus elaphus *and to evaluate the molecular prevalence of *Borrelia *spp., *Babesia *spp., *Theileria *spp., *Anaplasma *spp. and *Ehrlichia *spp. in ticks (*I. ricinus*) and tissues (heart, spleen, liver) by PCR. In September and October 2012 samples from 53 animals (24 bred in captivity; 29 wild) were collected. All animals originated from Harghita County that harbours 10% of red deer population from Romania. *Ixodes ricinus *was the dominant tick species (99.7%). Only three specimens of *Dermacentor marginatus *were identified. Samples (1007 ticks, 19 heart tissues, 12 spleen tissues and 21 liver tissues) were used for molecular diagnosis. Genomic DNA extraction was performed individually on all ticks and tissue samples, followed by PCR. In ticks samples, all investigated pathogens were present: *Anaplasma *spp. (6.5%), *Ehrlichia *spp. (2%), *Theileria *spp. (0.8%), *Babesia *spp. (0.6%) and *Borrelia *spp. (0.3%). From both heart and spleen tissue samples 3 pathogens were diagnosed: *Anaplasma *spp. (21%), *Theileria *spp. (15.8%), *Ehrlichia *spp. (5.3%) and *Anaplasma *spp. (16.7%), *Theileria *spp. (16.7%), *Babesia *spp. (8.3%), respectively. Only *Theileria *spp. (9.5%) and *Ehrlichia *spp. (4.8%) were present in liver samples. To our knowledge this is the first molecular study that evaluates the presence of tick-borne pathogens in ticks and tissues collected from *Cervus elaphus *in Romania, offering important eco-epidemiological data from public health perspective, stressing out the necessity of permanent surveillance.

This research was partially supported by grant CNCSIS IDEI PCCE 7/2010.

